# Introduction to the Special Issue: Microbiological Safety and Quality of Foods

**DOI:** 10.3390/foods11050673

**Published:** 2022-02-25

**Authors:** Alyexandra Arienzo, Valentina Gallo, Chiara Fanali, Giovanni Antonini

**Affiliations:** 1 National Institute of Biostructures and Biosystems (INBB), Viale delle Medaglie d’Oro 305, 00136 Rome, Italy; alyexandraarienzo@gmail.com; 2 Department of Science, Roma Tre University, Viale Guglielmo Marconi 446, 00146 Rome, Italy; valentina.gallo3@uniroma3.it; 3 Department of Science and Technology for Humans and the Environment, Campus Bio-Medico University of Rome, Via Álvaro del Portillo 21, 00128 Rome, Italy

## Introduction

Recent shifts in food production, processing and distribution, linked to the globalization of the food trade and the need to meet new consumers habits, are continuously challenging global food systems. Every effort is being made to ensure healthy and safe, food that is crucial to guarantee public health and wellbeing.

Despite the advancements in food safety management, foodborne diseases (FBD) still remain an important problem worldwide, with a significant negative impact on human health and countries’ economies and development. It has been determined that food unsafe for consumption causes 600 million cases of FBD every year, and 25% of all foods produced globally are lost due to microbial spoilage. Serious outbreaks have occurred, involving both developing and industrialized countries, showing how food safety is a transnational challenge and that a strong joint commitment between food safety authorities is needed.

Despite this awareness, the full extent of the impact of food contamination is still unknown. Foodborne contaminants are numerous, including viruses and bacteria, parasites, chemicals, toxins and allergens that cause a wide range of conditions. Globally, FBD caused by bacteria are more common than those caused by viruses and parasites. [1]. Moreover, between one-third and one-half of all human infectious diseases have a zoonotic origin. Among bacteria, *Campylobacter*, followed by *Salmonella*, are the major etiological agents of FBD, while, among viruses, *norovirus* is the foremost enteric pathogen of foodborne disease worldwide [2]. The most common foodborne parasites instead are protozoa such as *Cryptosporidium* spp., *Giardia intestinalis* and *Toxoplasma gondii*; roundworms such as *Trichinella* spp. and *Anisakis* spp.; and tapeworms such as *Diphyllobothrium* spp. and *Taenia* spp. [3]. In particular, Anisakiasis is an emerging zoonosis caused by the fish parasitic nematode *Anisakis.* Humans are accidental hosts that become infected by eating raw or undercooked fish that contain viable *Anisakis* spp. larvae.

The major determinants for the incidence of FBD are unsafe raw food, abused temperature, inadequate storage, improper handling, undercooking and cross contamination [4]. Food from animal sources, fresh produce and ready-to-eat (RTE) foods are the most at risk. In particular, RTE foods are an emerging issue concerning food safety. Furthermore, they have been demonstrated to contain antimicrobial-resistant strains. Since these products are consumed without any further treatment, they could serve as a vector for the spread of antibiotic-resistant microorganisms, posing a significant threat to public health [5].

**Figure 1:**
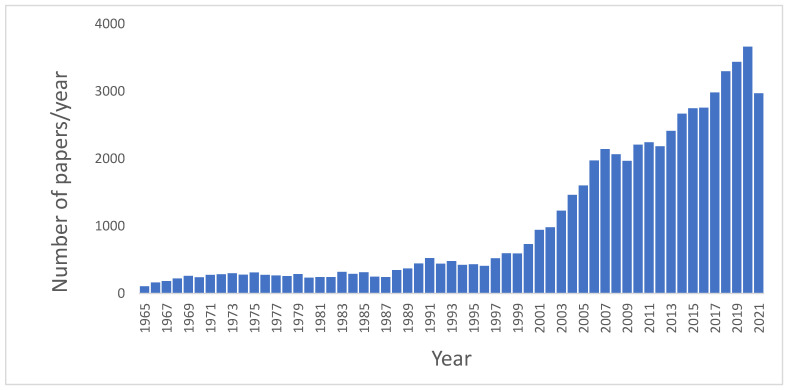
Number of papers per year (from 1965 to 2021) published regarding microbiological food safety.

The importance of these topics is documented by the increasing number of papers published related to Food Safety. In a basic search using PubMed database, from 1945 to 2021, selecting as the search topic “Microbiological food safety”, a total of 54,210 results were obtained. Although the first articles concerning microbiological food safety date from 1946, and since then a dozen articles appear in the following years, it is not until 1965 that a significant number of articles are published every year. Figure 1 shows the evolution of the number of papers per year (from 1965 to 2021) published regarding Microbiological food safety. As can be seen in this figure, approximately 80% of these papers have been published in the last 20 years, and the number continues to rise, revealing an increasing and ever-present interest towards the topic addressed in this Special Issue.

Microbiological food safety has been historically mainly studied in food from animal sources, as reflected in the number of papers found when the words “meat” (13,793 papers) and “dairy” (4189) were added as search criteria. However, starting from 2020, the interest in the Microbiological food safety of vegetables (3193 papers) has been rapidly increasing, as has the interest in ready-to-eat foods (1391); moreover, an important part of research is focusing on the topic “antibiotic resistance” (4754).

In this Special Issue, the original papers published address all these aspects, providing further insights on this topic.

Interesting results that bring to light the issues concerning unsatisfactory levels of microbiological contamination of food intended for consumption came from the first systematic nationwide analysis of food safety conducted in Lebanon by Kharroubi et al. [6]. During the campaign launched by the Lebanese MoPH, in the period from 2015 to 2017, 11,625 food samples were randomly collected in unannounced inspections in different food facilities, with 28.7% of them resulting unacceptable for consumption. Raw matrices such as red meat and poultry together with dairy products and spices displayed higher rejection rates. As for pathogens, *E. coli*, *Staphylococcus aureus* and *Salmonella* were detected in several samples, highlighting an unacceptable risk for consumers.

Always in Lebanon, Kassem et al. [7] investigated microbiological quality of raw minced beef, revealing an alarming occurrence of fecal coliforms and *E. coli* in the analyzed samples. Moreover, the authors investigated the antibiotic resistance profile of *E. coli* isolates, exposing the presence of many multi-drug-resistant strains.

Positive results come instead from the work of Aduah et al. [8], which studied the prevalence of antibiotic-resistant *Salmonella enterica* in RTE meats in Ghana and investigated the food safety knowledge among food vendors and consumers. The study revealed that 98% of the RTE meats were satisfactory and safe to eat regarding *Salmonella* and highlighted how these results are certainly linked to the good knowledge of meat safety and the proper handling practices of meat vendors and consumers.

Schill et al. [9] studied the microbiological and sensory quality of cultivated mushrooms available at the Austrian retail level on the day of purchase and after storage. The majority of mushrooms displayed high microbiological and sensory quality at the day of purchase while differences in the microbial load and sensory characteristics have been observed especially in re-packed mushrooms with long transport distances. The shelf life of these products appears to be affected by various aspects, including raw material quality, the processing environment and postharvest and storage conditions, and studying these intrinsic and extrinsic factors is important to guarantee safe products that maintain their microbiological and sensory qualities throughout shelf life.

Unsatisfactory results were instead obtained by Arienzo et al. [10], who analyzed the microbiological quality of ready-to-eat leafy green salads, sold in widespread supermarket chains in Lazio, Italy, during shelf-life and home-refrigeration. The study revealed high, unsatisfactory total bacterial loads in all analyzed samples on the packaging date and expiry date and a worrying prevalence of *Salmonella* spp. (67%) regardless of the selected varieties and cost categories.

An interesting study has been conducted by Maio et al. [11], who evaluated the microbiological quality of different food products sold in supermarkets at the expiry date. It was observed that 70.21% of the samples analyzed at the expiry date failed in at least one microbiological criterion, resulting, however, as safe given the absence of pathogenic microorganisms. These data point out the issues concerning shelf life and the challenges that food manufacturers face in order to minimize decrease in product quality due either to spoilage by bacteria or to biochemical processes.

As mentioned above, apart from microbiological risk, pluricellular parasites (e.g., nematodes) also represent an important public health issue that affect food quality and safety. Ahuir-Baraja et al. [12] have evaluated the effectiveness of gutting Blue Whiting in Spanish supermarkets as an Anisakidosis safety measure. The results show that the prevalence of ascaridoid larvae infection found in ungutted blue whiting was considerably high. However, if gutting reduces the larval burden, it cannot be considered an effective method for the total removal of ascaridoid larvae. Larger and heavier fish present higher infection levels. The time until evisceration is also an important factor.

These results confirm the need to develop and improve new strategies to achieve safe and healthy food; currently, in this context, novel technologies are being investigated in many different countries. Alternative agricultural practices, such as hydroponic culture, represent an interesting example. Hydroponic vegetables are cultivated in the absence of soil and rely on the use of balanced nutrient solutions to support their development. Given the more controlled environment and the feasibility, hydroponic culture is an attractive alternative to increase the quality and safety of produce. Currently, the literature lacks significant reports on the benefits of this technique, but there is a general belief that it can provide healthy and more hygienic products, and for this reason, it is becoming more and more popular. In the work by Lam et al. [13], this issue has been addressed. Researchers investigated the microbiological contamination and the presence of antibiotic resistant bacteria in hydroponic lettuce, compared to conventional and organic lettuce in retail. As expected, hydroponic lettuce contained the least number of resistant bacteria among the three groups. However, antibiotic-resistant bacteria contamination also appears to be an issue not to be underestimated in this particular type of product, and water appears to be the crucial element to control in order to obtain microbiologically safe products and reduce the spread of AMR.

Alternatives to conventional processing procedures are also taking place. An alternative to classic thermal sterilization of liquid foods is the use of ultrasounds. This technique has been applied primarily to milk and fruit juices, with the aim of increasing shelf life due to the reduction of contaminating microorganisms, still improving the quality of products, that display increased levels of antioxidants and bioactive compounds. Ma et al. [14] studied the effect of ultrasound-combined sterilization technology on the safety and quality of grape juice. Ultrasound treatment is often more effective when combined with moderate heat and is known as thermosonication. These authors found that thermosonication (TS) and TS combined with nisin (TSN) are very promising alternatives to conventional thermal sterilization; they are able to improve the nutritional and functional characteristics of grape juice, increasing the total phenolic content and antioxidant capacity, and at the same time ensure microbiological safety.

In addition, alternative rapid, sensitive, specific and cost-effective microbiological methods are being developed and studied in order to help industries obtain reliable results faster and more efficiently, possibly directly on site, in order to take quick corrective actions and, therefore, improve food safety management. In their work, Marri et al. [15] carried out the validation of a rapid alternative method for the assessment of microbiological quality in raw milk, namely, the Microbiological method (MBS), a colorimetric, culture-based system that allows one to perform microbiological analyses in the absence of either dedicated facilities or specialized personnel. The study demonstrates the accuracy of this alternative method for the determination of total viable bacterial count in cow’s raw milk and shows its potential as a reliable tool for dairy industries to promptly evaluate the safety and quality of raw ingredients or to evaluate the effectiveness of sterilization procedures.

Taken together, these studies are clear evidence of how the achievement of safe and healthy food is still a complex and multifaceted process. Food manufacturers have to meet consumer demands for freshness and convenience without compromising the safety and shelf life of foods, and the food industry is thus continuously challenged and seeking sustainable and practical methods to ensure safety of products and guarantee the maximum level of security for consumers. The studies included in this Special Issue are clear evidence of the importance of prioritizing food safety by raising awareness among consumers and business owners, promoting food safety policies, implementing national surveillance systems and investing in the education and training of qualified resources, as well as infrastructures. Together, these papers place renewed emphasis on the need for a well-established and reliable surveillance and monitoring system to ensure food safety and reduce the spread of antimicrobial resistance.
